# *Ganoderma lucidum*: Current advancements of characteristic components and experimental progress in anti-liver fibrosis

**DOI:** 10.3389/fphar.2022.1094405

**Published:** 2023-01-09

**Authors:** Haoyuan Peng, Lei Zhong, Lin Cheng, Lu Chen, Rongsheng Tong, Jianyou Shi, Lan Bai

**Affiliations:** ^1^ The State Key Laboratory of Southwestern Chinese Medicine Resources, Department of Pharmacy, Chengdu University of Traditional Chinese Medicine, Chengdu, China; ^2^ Department of Pharmacy, Personalized Drug Therapy Key Laboratory of Sichuan Province, Sichuan Provincial People's Hospital, School of Medicine, University of Electronic Science and Technology of China, Chengdu, China; ^3^ College of Medicine, Southwest Jiaotong University, Chengdu, Sichuan, China

**Keywords:** *Ganoderma lucidum*, liver fibrosis, triterpenes, polysaccharide, chromatography, pharmacology

## Abstract

*Ganoderma lucidum (G. lucidum, Lingzhi)* is a well-known herbal medicine with a variety of pharmacological effects. Studies have found that *G. lucidum* has pharmacological effects such as antioxidant, antitumor, anti-aging, anti-liver fibrosis, and immunomodulation. The main active components of *G. lucidum* include triterpenoids, polysaccharides, sterols, peptides and other bioactive components. Among them, the triterpenoids and polysaccharide components of *G. lucidum* have a wide range of anti-liver fibrotic effects. Currently, there have been more reviews and studies on the antioxidant, antitumor, and anti-aging properties of *G. lucidum*. Based on the current trend of increasing number of liver fibrosis patients in the world, we summarized the role of *G.lucidum* extract in anti-liver fibrosis and the effect of *G. lucidum* extract on liver fibrosis induced by different pathogenesis, which were discussed and analyzed. Research and development ideas and references are provided for the subsequent application of *G. lucidum* extracts in anti-liver fibrosis treatment.

## 1 Introduction

*Ganoderma lucidum* is the dried fruiting entity of *G. lucidum (Leyss. ex Fr.) Karst.* or *Ganoderma purpurea* Zhao, Xu et Zhang, a fungus of the family Polyporaceae, which are one of the most well-known kinds of therapeutic fungi in China and a very representative large species of Chinese herbal medicine (Wang et al., 2017). *G. lucidum* has been utilized for over 2,000 years in China and has been recorded in Shennong Ben Cao Jing (Eastern Han Dynasty), Baopu Zi—Immortal Medicine (Eastern Jin Dynasty), Compendium of Materia Medica (Ming Dynasty), the Pharmacopoeia of the People’s Republic of China (2000 edition), etc., (Luo et al., 2021) in various ancient books and modern standards. With both medicinal and edible properties, *G. lucidum* has been considered to have broad development prospects. In 2020, the State Administration for Market Regulation (China) included healthy food products such as *G. lucidum* in the raw material catalog (Luo et al., 2021), indicating that *G. lucidum* occupies an important position in the health food market in China. Meanwhile, *G. lucidum* has been added to the US Pharmacopoeia and the European Pharmacopoeia, indicating that *G. lucidum* is also widely used internationally. The bioactive substances of *G. lucidum* include polysaccharides, triterpenes, sterols, peptides, and so on. The pharmacological effects of *G. lucidum* include antioxidant (Ferreira et al., 2009), antitumor/anticancer (Moradali et al., 2007), antimicrobial (Barros et al., 2007), immunomodulatory (Borchers et al., 2004), anti-inflammatory (Moro et al., 2012), antiatherogenic (Mori et al., 2008), and hypoglycemic effects (Hu et al., 2006). In recent years, a growing number of research have discovered that Ganoderma has multiple hepatoprotective benefits on different liver injuries, including alcoholic liver disease, viral hepatitis, autoimmune hepatitis, non-alcoholic fatty liver disease (NAFLD), hepatitis B, inflammation, fibrosis, and cholestatic liver diseases (Aydın and Akçalı, 2018).

The number of people with liver fibrosis is currently on the rise worldwide, and there is an urgent need to develop preventive and therapeutic measures against liver fibrosis. Therefore, in this paper, we searched databases such as PubMed and Web of Science for keywords such as *G. lucidum*, liver fibrosis, *G. lucidum* polysaccharides, and *G. lucidum* triterpenes for the discussion. Firstly, we summarized the reported anti-fibrotic components of *G. lucidum*. Secondly, we outlined the anti-fibrotic effects of *G. lucidum* extracts according to different pathogenic models and influencing factors of liver fibrosis. Finally, the discussions of existing studies showed the possible research directions that were proposed to provide research ideas and references for further development of the application of *G. lucidum* in anti-liver fibrotic diseases.

## 2 The main anti-fibrosis ingredients of *G. lucidum*

Fibrosis of the liver is a reversible liver disease. Modern research has found that *G. lucidum* encompasses *G. lucidum* polysaccharides, triterpenes, and other bioactive components, which have apparent anti-liver fibrosis pharmacological effects (Zhan et al., 2015; Xu et al., 2016). We summarized the specific components of *G. lucidum* anti-liver fibrosis reported in the literature so far, almost all of them are triterpenoids, and extracts of *G. lucidum* polysaccharides have also been reported to have anti-liver fibrosis effects, but the isolation of monomer components remains to be studied (Table 1).

**TABLE 1:** The main anti-fibrosis ingredients of *Ganoderma lucidum*.

Number	Category	Compound name	Ganoderma species	References
1	Triterpenes	Ganoderic acid A	*G. lucidum*	El-Mekkawy et al. (1998)
2	Triterpenes	Ganoderic acid B	*G. lucidum*	Kubota et al. (1982)
3	Triterpenes	Ganoderic acid C	*G. lucidum*	Seo et al. (2009)
4	Triterpenes	Ganoderic acid D	*G. lucidum*	Qiao et al. (2007)
5	Triterpenes	Ganoderic acid F	*G. lucidum*	Yang et al. (2012)
6	Triterpenes	Ganoderic acid G	*G. lucidum*	Kikuchi et al. (1985)
7	Triterpenes	Ganoderic acid H	*G. lucidum*	Yang et al. (2012)
8	Triterpenes	Ganoderic acid DM	*G. lucidum*	Adams et al. (2010)
9	Triterpenes	Ganoderic acid X	*G. lucidum*	Li et al. (2009)
10	Triterpenes	Lucidone A	*G. amboinense*	Gan et al. (1998)
11	Triterpenes	Lucidone B	*G. lucidum*	Nishitoba et al. (1985)
12	Triterpenes	Lucidone C	*G. lucidum*	Nishitoba et al. (1986)
13	Triterpenes	Lucidone D2	*G. lucidum*	Nishitoba et al. (1986)
14	Triterpenes	Ganoderal A	*G. lucidum*	Niedermeyer et al. (2005)
15	Triterpenes	Ganoderal B	*G. lucidum*	Nishitoba et al. (1988)
16	Triterpenes	Ganoderma lactone A	Ganoderma sp	Lakornwong et al. (2014)
17	Triterpeness	Ganoderma lactone D	Ganoderma sp	Lakornwong et al. (2014)
18	Triterpenes	Ganoderma lactone F	Ganoderma sp	Lakornwong et al. (2014)
19	Triterpenes	Ganoderma lactone G	Ganoderma sp	Lakornwong et al. (2014)
20	Triterpene	12-Hydroxy G-A C2	*G. lucidum*	Yang et al. (2007)
21	Triterpene	20-Hydroxy L-A A	*G. lucidum*	Ma et al. (2002)
22	Triterpene	20-Hydroxy L-A D2	*G. lucidum*	Akihisa et al. (2005)
23	Triterpene	20-Hydroxy L-A E2	*G. lucidum*	Akihisa et al. (2005)
24	Triterpene	20-Hydroxy L-A F	*G. lucidum*	Akihisa et al. (2005)
25	Triterpene	20-Hydroxy L-A N	*G. lucidum*	Akihisa et al. (2005)

*G. lucidum* triterpenes were found to have significant inhibitory proliferative effects on platelet-derived growth factor (PDGF)-BB-stimulated HSC-T6 (rat HSC) cell lines. 25 μg/mL *G. lucidum* triterpenes inhibited HSC-T6 cell proliferation and triggered apoptosis. Meanwhile, the phosphorylation of cell cycle proteins D1, D2, and PDGFβR was inhibited, while the phosphorylation of *ß* was enhanced. Thus, the expression of α-SMA was inhibited. *G. lucidum* triterpene extract may inhibit the multiplication of PDGFβR-activated hepatic stellate cells by preventing the phosphorylation of platelet-derived growth factor, thus showing its effect against liver fibrosis (Wang et al., 2009; Qiu et al., 2019). *G. lucidum* triterpenes exerts anti-fibrotic effects on liver fibrosis through several mechanisms. They inhibited HSC proliferation and upregulated collagenase expression, thus inhibiting collagen deposition; *G. lucidum* was anti-oxidant activity, on the other hand, is crucial to its hepatoprotective impact (Qiu et al., 2019). These two methods combined successfully to slow the development of liver fibrosis (Wang et al., 2009; Qiu et al., 2019).

**FIGURE 1:**
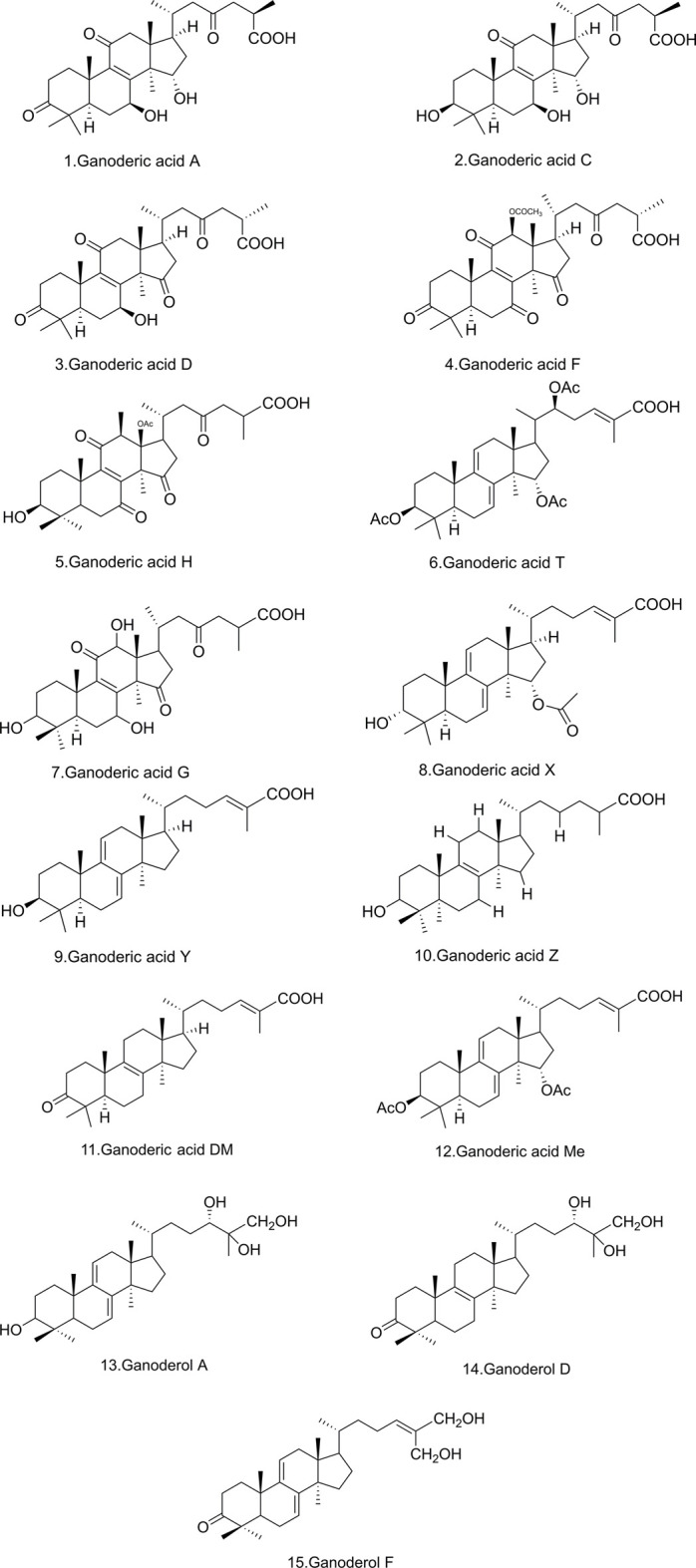
Chemical structure of the main active components of Ganoderma triterpenes.

Several highly oxidized and pharmacologically active triterpenoids can be extracted from *G. lucidum* at present. *G. lucidum* acids were the primary source of pharmacological activity of *G. lucidum*; on the contrary, their triterpenoids containing carboxyl groups were generally called *G. lucidum* acids, which are highly oxidized derivatives of lanolin (Satria et al., 2019). These substances have complicated structures, high molecular weights, and high lipophilicity (Lin et al., 2003). Their main chemical structures are shown in Figure 1. The triterpenoids found naturally in Ganoderma originated from the intermediate wool sterol backbone. The cyclization of squalene-2,3-epoxide gives protosterol, a carbon-cationic intermediate that undergoes a further skeleton rearrangement, produces a tetracyclic wool sterol (C_30_H_54_) skeleton. Tetracyclic wool sterols play the role of intermediate molecules in the biosynthesis of various wool sterane triterpenes. The triterpenoids were uncommon secondary metabolites in the genus Ganoderma and were the products of side-chain degradation of wool sterane-type triterpenoids. The common triterpenoids in the genus Ganoderma have a carbon skeleton of 24 or 27 carbon atoms (Koo et al., 2021). The activity relationship analysis of triterpenoids isolated from *G. lucidum* revealed that the type of side chain, the C-3 carbonyl group, the number of double bonds, and the number of hydroxyl groups have a crucial impact in cytotoxicity (Wu et al., 2013).

Ganoderic acid’s action may be mostly attributable to the hydroxylation of its fuzzy sterane triterpene structure. As illustrated in Figure 1, Ganoderic acid A (GA-A) is hydroxylated at positions 7 and 15, while Ganoderic acid H (GA-H) is hydroxylated at C-3, and inactive Ganoderic acid F (GAF) is not hydroxylated. Other triterpenes have hydroxyl or acetoxy groups at positions 3, 7, and 15, including Ganoderic acid C1 (GA-C1), Ganoderic acid C2 (GA-C2), and Ganoderic acid C3 (GA-C3) (GA-C2) (Siwulski et al., 2015), Ganoderic acid D (GA-D), Ganoderic acid T (GA-T) (Tang et al., 2006), Ganoderic acid X (GA-X), Ganoderic acid Y (GA-Y) (Hajjaj et al., 2005), Ganoderol A (Liu et al., 2006), Ganoderol B (Hajjaj et al., 2005), Ganoderol B (Liu et al., 2006), and Ganoderol glycol (Liu et al., 2006), were also shown to be inhibitors (Jiang et al., 2008). The structure and anti-liver fibrosis effect of *G. lucidum* triterpene extracts after isolation is also an urgent need for development and research nowadays.

## 3 Pathology, signal pathways and experimental models of hepatic fibrosis

**FIGURE 2:**
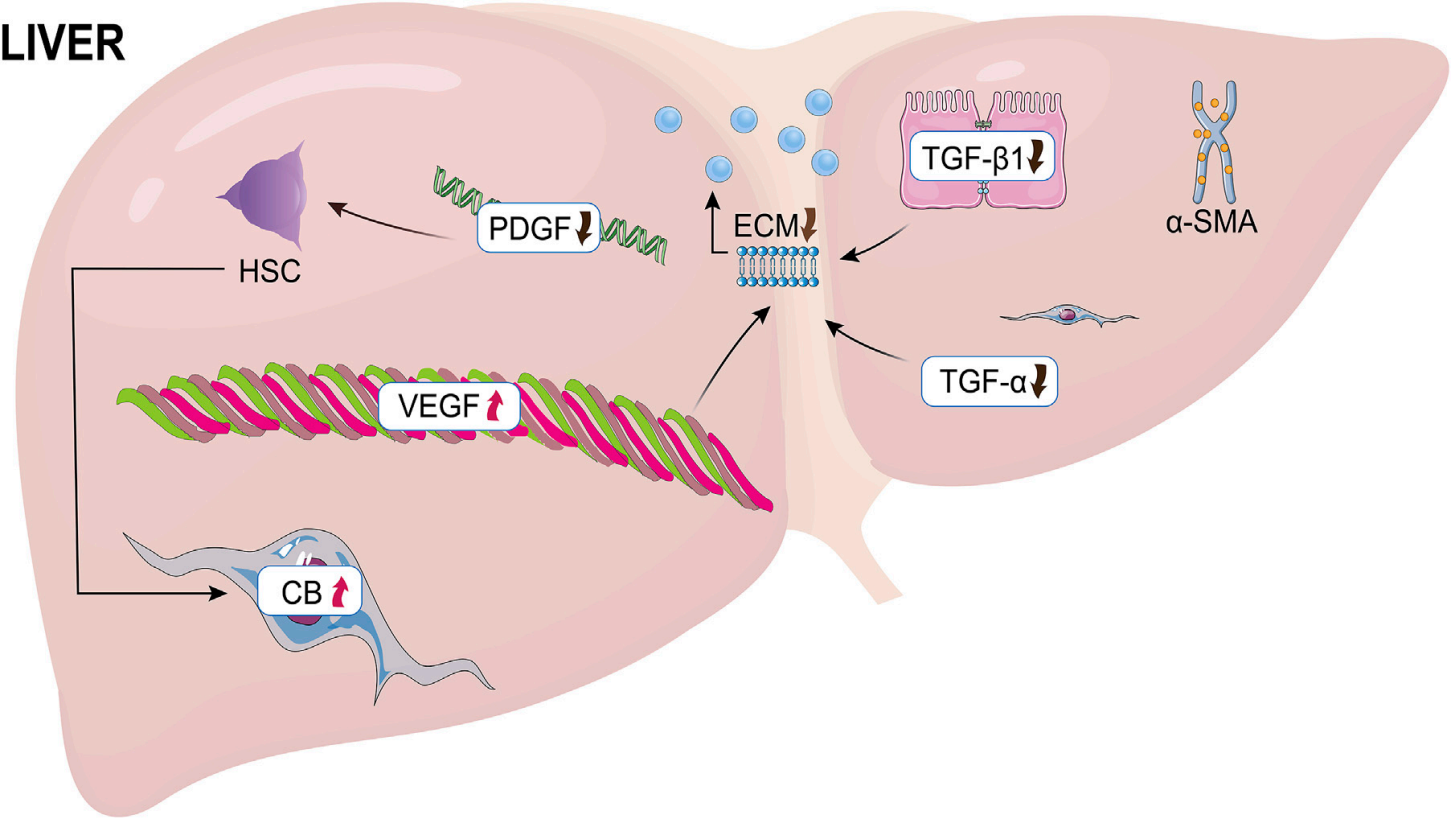
The causes of liver fibrosis. HSC, hepatic stellate cells; PDGF, Platelet-derived growth factor; ECM, extracellular matrix; VEGF, vascular endothelial growth factor; CB, cannabinoid.

Hepatic fibrosis formation demands the stimulation and proliferation of hepatic stellate cells (HSCs), as well as the accumulation of extracellular matrix (ECM), and simultaneous creation of α-smooth muscle actin (α-SMA) and type I collagen. PDGF-BB homodimers are strong ligands for PDGF receptors (PDGFRs), hence boosting the expansion of HSC. Inhibiting the activation and proliferation of activated HSCs, as well as inducing their death, are considered therapeutic methods for the prevention and therapy of liver fibrosis (Wang et al., 2009). Moreover, fibrogenesis is triggered by the activation and proliferation of myofibroblasts, which are the main supply of ECM in wounded livers (Bataller and Brenner, 2005; Kisseleva and Brenner, 2008). In fibrotic livers, activated hepatic stellate cells (aHSCs) were the main source of myoblasts, although they are one of their precursors. Endogenous portal fibroblasts, fibroblasts, bone marrow-originating cells, and myofibroblasts produced from hepatic parenchymal cells go through the epithelial-mesenchymal transition (EMT) and produce a large number of myofibroblasts in fibrotic livers. According to the cause of liver fibrosis, several types of cells trigger myofibroblasts (Iwaisako et al., 2014). In the resting state, hematopoietic stem cells, called resting hematopoietic stem cells, are responsible for storing vitamin A in the liver. Hematopoietic stem cells are activated by inflammatory mediators due to liver injury, which in turn differentiate into myofibroblasts (Zhang et al., 2016). Thus, tissue remodeling in the liver is begun by ECM proteins and matrix metalloproteinases (MMPs) released by hematopoietic stem cells (Puche et al., 2013; Li et al., 2015). HSC proliferation is also boosted by growth factors like TGF-α and epidermal growth factor (Meyer et al., 1990). A healthy liver includes collagen IV and collagen VI in the Disse area. During fibrosis, however, they are exchanged for collagens I and II and fibronectin (Brown et al., 2006). TGF-β1 is normally dormant, but upon excitation, it triggers a signaling pathway including Smad proteins that results in the creation of collagen. In addition, TGF-β1 promotes the transformation of dormant hematopoietic stem cells into ECM-secreting myofibroblasts (Breitkopf et al., 2006). Also, the initiation of hepatic angiogenesis is recognition of the vascular endothelial growth factor receptor (VEGF). Overall, these expansion factors induce ECM remodeling, leading to collagen synthesis (Schuppan et al., 2001). In liver fibrosis, neurochemical and neurotrophic substances also have an influence on HSCs. The neuroendocrine system is upregulated by liver damage, and stimulated HSCs begin to display receptors that govern cannabinoid (CB) signaling (Figure 2) (Mukhopadhyay et al., 2010).

**FIGURE 3:**
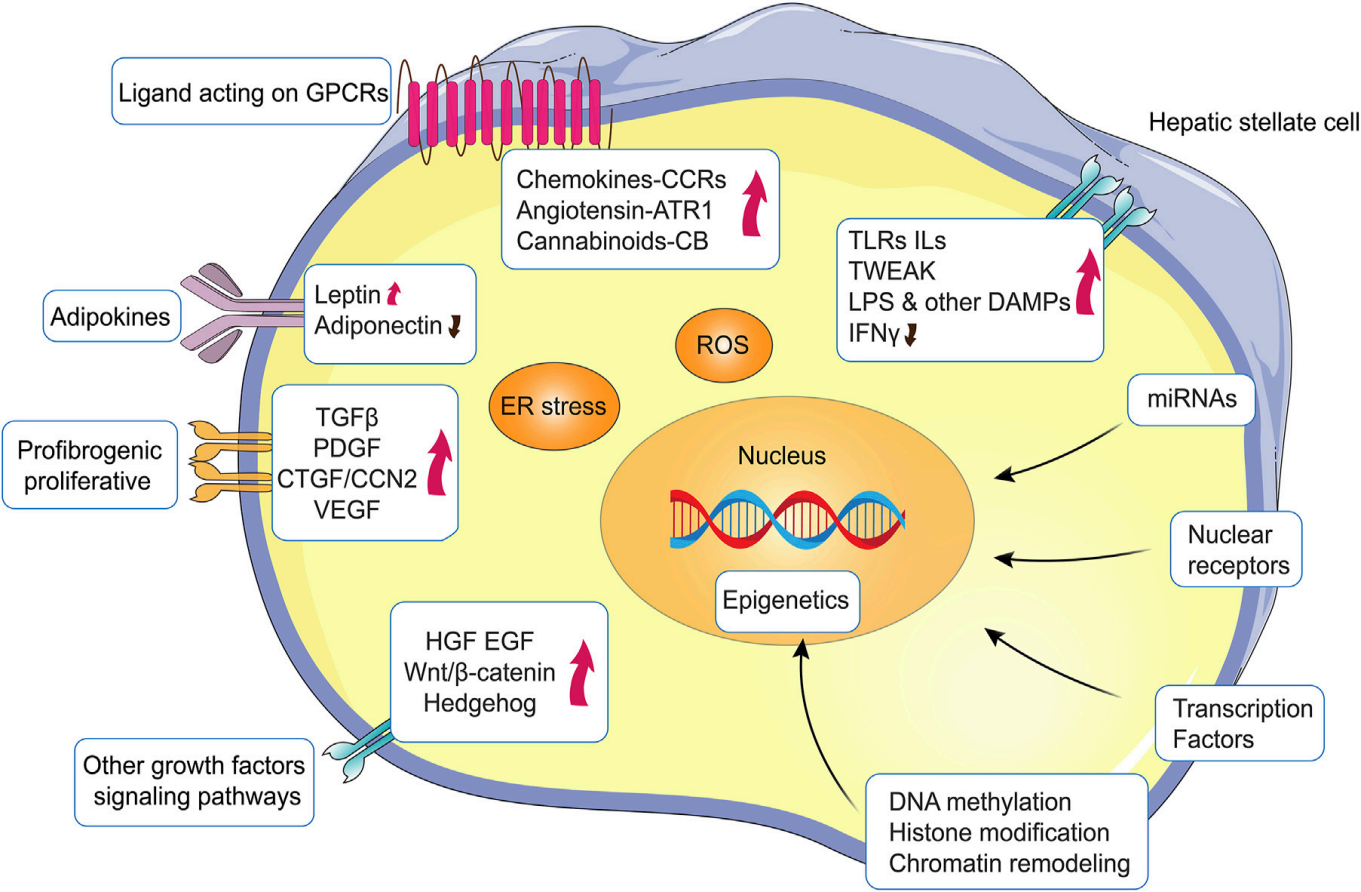
Major signaling pathways, molecules and mechanisms regulating HSC activation. HSC activation is regulated by a multitude of pathways and signaling molecules or events that can either sustain or inhibit HSC activation and subsequent proliferative and/or profibrogenic responses.

HSC activation may also be dependent on contact with altered ECM (shifting from basal-like to fibrillary ECM) *via* integrin-mediated signals (Henderson et al., 2013), to promote HSC activation *via* peptide mediators (PDGF, FGF, HGF, VEGF) that stay trapped in the altered ECM (Lee et al., 2015). By interacting with their cognate receptor(s), multiple peptide growth factors can affect and sustain one or more of the phenotypic responses of activated HSC (Figure 3) (Parola and Pinzani, 2019). A typical example is the signaling pathways elicited by TGβ1 or PDGF, growth factors that act on myofibroblasts but are also released by these cells. Similar considerations can be made for other ligand-receptor-induced signaling pathways, such as those involving HGF, EGF/EGFR, VEGF/VEGFR, Wnt/β-catenin, Hedgehog, endotelins, cannabinoids, adipokines, retinoid and vitamin D receptors, integrins, and TLRs (Higashi et al., 2017). As a pertinent example, connective tissue growth factor (CTGF) is believed to be crucial in mediating TGFβ1 pro-fibrogenic effects (Jun and Lau, 2011) and experimental targeting of CTGF can impact HSC activation and suppress experimental fibrosis (Hao et al., 2014). Another example is the use of losartan, an inhibitor of the angiotensin II receptor ATR1, which is strongly expressed by activated HSC, with angiotensin II boosting proliferation, migration, contractility, and TGFβ1 and collagen I production in these cells (Moreno and Bataller, 2008). Losartan has been shown in animal studies (Moreno et al., 2010) and maybe in hepatitis C virus (HCV) patients (Salama et al., 2016) to prevent fibrosis *via* modulating non-phagocytic NADPH-oxidase and profibrogenic genes (Colmenero et al., 2009).

HSC express a number of nuclear transcription factor receptors, including PPAR-γ and PPAR-δ, farnesoid X receptor (FXR), liver X receptor (LXR), vitamin D receptor (VDR), nuclear receptor subfamily 4 group A member 1 (NR4A1), and nuclear receptor subfamily 1 group D member 1 (REV-ERB) (Tsuchida and Friedman, 2017). These nuclear receptors, which control energy fluxes and metabolic pathways, are dysregulated in chronic liver diseases (CLD), especially in progressive NAFLD (Wang et al., 2015), and have been shown to limit HSC activation and fibrosis development.

*G. lucidum* anti-fibrotic activity could also result from the enhancement of collagenase (CLG), as therapy with *G. lucidum* extracts (GLE) decreased the gene expression of collagen (α1)(I), smooth muscle α-actin, metalloproteinase tissue inhibitor, and metalloproteinase-13 in liver fibrosis-induced rats (Wu et al., 2010). Consequently, decreasing hepatic hydroxyproline (HYP) concentration and enhancing liver histology, GLE restored thioacetamide (TAA)-induced reduction in collagenase activity and enhanced collagen clearance (Wu et al., 2010; Qiu et al., 2019). Several substances are known to produce liver fibrosis and are therefore frequently employed to develop animal models for the research of this specific kind of lesions. For most cases, intraperitoneal administration of these substances causes liver fibrosis within a comparatively brief time frame (Smith, 2013). When taken orally or by inhalation, the development of fibrosis is restricted and delayed. Popular as a result of their great repeatability, convenience of use, and accurate portrayal of the pathways participating in human liver fibrosis, these chemically-based animal models are widely utilized (Crespo Yanguas et al., 2016). Therefore, we summarized the model of liver fibrosis caused by d-galactosamine, ethanol, CCl_4_, high-fat food, and formaldehyde and the factors influencing the anti-liver fibrosis experiment with *G. lucidum* (Table 2).

**TABLE 2:** Anti-hepatic fibrosis model of *G. lucidum* and the factors affecting it.

Pretreatment and liver fibrosis inducer	Influence parameter
D-Galactosamine	AST, ALT, SOD
Ethanol	MDA
CCl_4_	GOT, GPT
High-fat food	TG, LDL
Formaldehyde	ALP, AST, ALT

AST, aspartate aminotransferase; ALT, alanine aminotransferase; SOD, superoxide dismutase; MDA, malondialdehyde; GPT, pyruvate aminotransferase; GOT, glutamate oxaloacetate transaminase; TG, triglycerides; LDL, low-density lipoprotein; ALP, alkaline phosphatase.

**FIGURE 4:**
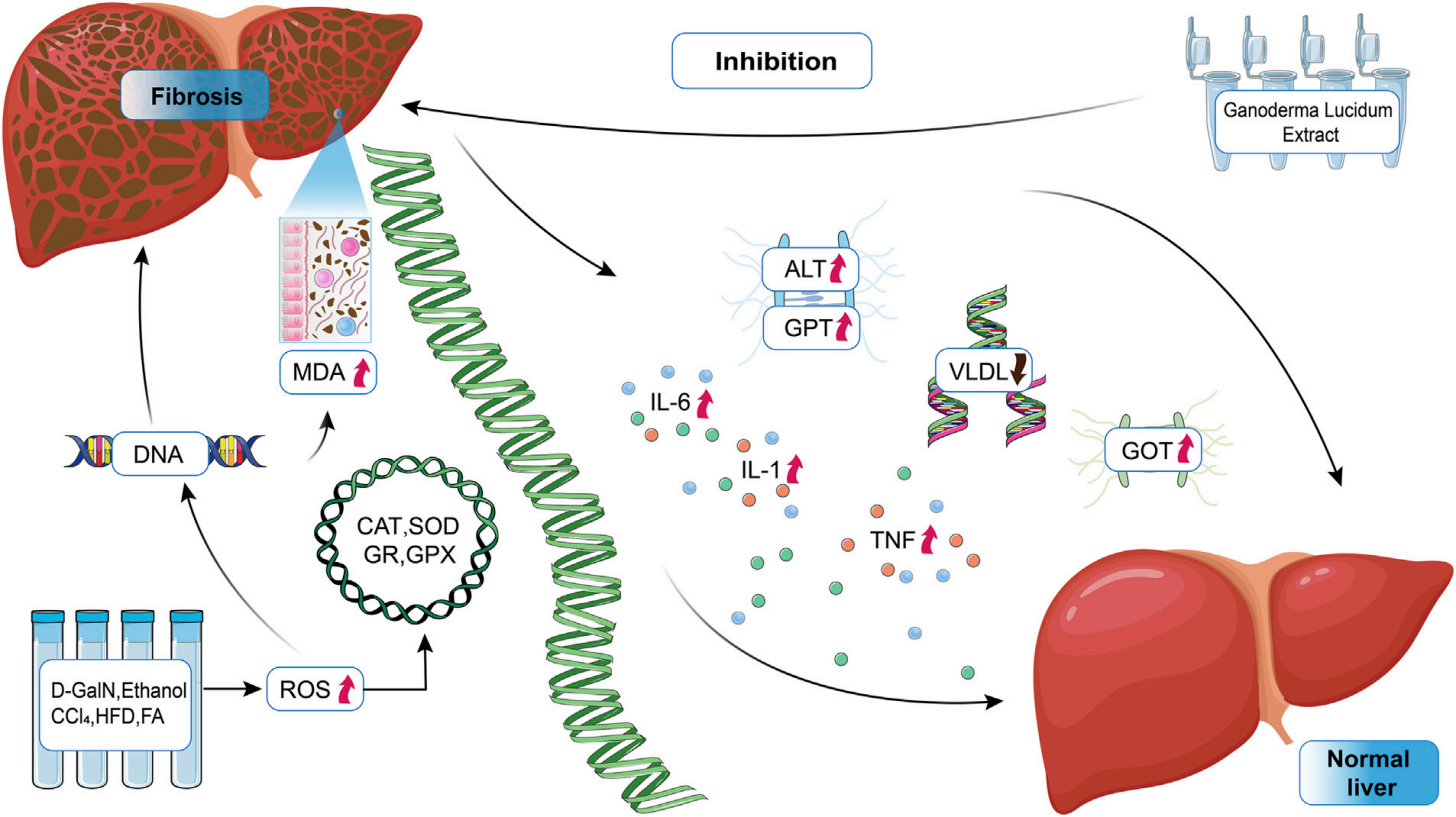
Mechanism of action of *Ganoderma lucidum* in the treatment of liver fibrosis. ALT, alanine aminotransferase; MDA, malondialdehyde; GPT, pyruvate aminotransferase; GOT, glutamate oxaloacetate transaminase; ROS, reactive oxygen species; CAT, catalase; GR, glutathione reductase; GPX, glutathione peroxidase; VLDL, very-low-density lipoprotein.

*G. lucidum* extracts could significantly increase the activity of certain enzymes or decrease specific indicators. We summarized the mechanisms that usually cause liver fibrosis, as shown in Figure 4, as well as the therapeutic effects of Ganoderma on them for the review summary and to provide ideas for subsequent studies.

## 4 Protective effect of *G. lucidum* on different liver fibrosis models in experiments

There are many causative factors of liver fibrosis, and the changes in the levels of the influencing factors in different liver fibrosis model experiments demonstrate the good anti-fibrotic effect of *G. lucidum*.

### 4.1 Protective effect of *G. lucidum* on D-galactosamine-induced hepatic fibrosis

The effect of total triterpenes extracted from *G. lucidum* on a model of experimental liver fibrosis induced by D-galactosamine (D-GalN) was extensively studied in mice (Shieh et al., 2001; Shi et al., 2008). *G. lucidum* triterpene extract (80 mg/kg) strongly prevented the elevation of blood alanine aminotransferase (ALT) and the hepatic models’ triglyceride levels, with outcomes comparable to malic acid, a reference material known for its protective benefits (Ala-Kokko et al., 1987). Oxidative stress was primarily caused by the manufacturing of reactive oxygen species (ROS), which was an imbalance between free radical exposure and antioxidant defenses. ROS also play an important role in cell proliferation and signaling (Zhu et al., 2020). Free radicals damage hepatocytes by directly damaged key biomolecules, included DNA, lipids, and proteins (Alía et al., 2003). *G. lucidum* triterpene extract also prevented the decline in superoxide dismutase (SOD) activity and glutathione (GSH) content and inhibited the increase in malondialdehyde (MDA) content in mice with D-galactosamine-induced liver fibrosis. It likewise improved histopathological changes.

D-GalN-induced liver fibrosis was characterized by a large rise in serum marker enzyme (AST, ALT) activity, liver MDA levels, and a significant decline in liver SOD and GSH activity (Shi et al., 2008). Pretreatment of mice with *G. lucidum* total triterpene extracted kept these parameters at their normal values. Histopathological examination of liver sections complemented these biochemical findings. From the biological parameters and the histopathological examination of the liver, it was inferred that the optimal hepatoprotective effect of *G. lucidum* total triterpene extracted was noticed following therapy at a dose of 180 mg/kg (Shi et al., 2008; Soares et al., 2013). The results might indicate that the triterpenoids isolated from *G. lucidum* had powerful defensive effects against D-galactosamine-induced liver fibrosis. *G. lucidum* triterpene extract’s hepatoprotective effect might be related to the activity of enzymes that neutralize free radicals, thus improving anti-oxidant capacity (Kim et al., 1999).

### 4.2 Protective effect of *G. lucidum* on alcoholic-induced hepatic fibrosis

Liver fibrosis caused by alcohol consumption is among the major risk factors for developing of many liver disorders. Alcohol misuse causes 10%–35% of alcoholic hepatitis and around 10% of cirrhosis (Stickel et al., 2017). *G. lucidum* had certain anti-oxidant effects on ethanolic liver fibrosis. It was postulated that among the primary causes of ethanol-induced liver fibrosis is free radical-induced lipid peroxidation, which is mostly caused by chronic alcohol consumption (Bautista and Spitzer, 1999; Meagher et al., 1999).

ICR mice were used to research the preventive effect of *G. lucidum* against ethanol-induced liver fibrosis and its mode of action (Shieh et al., 2001). It has been disclosed that ethanol increases lipid peroxidation in the liver (Kera et al., 1985). It was also shown that *G. lucidum* prevented ethanol-induced lipid peroxidation by 95% in a dose-dependent way. *G. lucidum* inhibited lipid peroxidation and dramatically reduced MDA production in the liver homogenates of control mice (untreated with ethanol), as shown in Table 3. These findings disclosed that *G. lucidum* was protective effect against ethanol-induced liver fibrosis was at least partially attributable to a reduction in MDA production. Their findings suggested that free radical generation might contribute to etiology of ethanol-induced liver damage and liver fibrosis. These effects may be attributable to its capacity to reduce membrane lipid peroxidation and free radical production or to scavenge free radicals (Table 4) (Bautista and Spitzer, 1999; Shieh et al., 2001).

**TABLE 3:** Effect of *G. lucidum* extracts (GL) on lipid peroxidation in mouse (untreated with ethanol) liver homogenates.

Groups	MDA (nmole/mg protein)	Inhibition rate (%)
Saline	0.162 ± 0.006	—
GL (10 mg/kg)	0.129 ± 0.02	20.37
GL (25 mg/kg)	0.125 ± 0.0005*	22.84
GL (50 mg/kg)	0.121 ± 0.008*	25.31

Data source: (Shieh et al., 2001) Each number indicates mean ± S.E., (*n* = 10). *p < .001, notably distinct from the standard control group. *p < .05, notably distinct from the standard control group. Analysis of variance with Dunnett’s test. *p*-values below .05 were considered significant.

**TABLE 4:** Inhibitory effect of *G. lucidum* extracts (GL) on ethanol-induced lipid peroxidation in mouse liver homogenates.

Groups	MDA (nmole/mg protein)	Inhibition rate (%)
Saline	0.046 ± 0.01	—
95% Ethanol (0.1 mL)	0.095 ± 0.01*	—
95% Ethanol (0.1 mL)+GL (10 mg/kg)	0.058 ± 0.03	38.9
95% Ethanol (0.1 mL)+GL (25 mg/kg)	0.048 ± 0.02	49.5
95% Ethanol(0.1 mL)+GL(50 mg/kg)	0.045 ± 0.01*	52.6

Data source: (Shieh et al., 2001) Each number indicates mean ± S.E., (*n* = 10). *p < .05, notably distinct from the standard control group. *p < .05, notably distinct from the ethanol group. Analysis of variance with Dunnett’s test. *p*-values below .05 were considered significant.

### 4.3 Protective effect of *G. lucidum* on CCl_4_-induced hepatic fibrosis

In the liver, cytochrome P450-dependent oxidases activate CCl_4_ to produce CCl_3_ radicals, which bind to cytosolic lipids and proteins under the influence of oxygen and trigger lipid peroxidation *via* hydrogen extraction (Kadiiska et al., 2000; Lim et al., 2000). These factors lead to alterations in the structure of the endoplasmic reticulum and other membranes, and loss of metabolic enzyme activity, which impairs liver function (Soares et al., 2013). And regards the activity of reducing elevated glutamate pyruvate aminotransferase (GPT) levels, *G. lucidum* treatment showed therapeutic activity, as shown in Table 5, where a single injection of CCl_4_ induced a significant increase in serum glutamate oxaloacetate transaminase (GOT) and GPT levels 72 h after intoxication against the control group (Lin et al., 1995). Lactate dehydrogenase (LDH) values were statistically significantly lower in the drug-treated group than in the CCl_4_-treated control rats, except in rats treated with *G. lucidum* (10 mg/kg). The results also indicated that Ganoderma showed potent hepatoprotective effects by observing a reduction in serum LDH levels (Lin et al., 1995). The targeted conjugates can protect mice, according to a preliminary biological review from acute liver fibrosis generated by carbon tetrachloride (Jin et al., 2014). The histological changes observed in the drug treatment group were smaller than those noticed inside the group. Administration of *G. lucidum* in an attempt to lessen the hepatotoxic effects of CCl_4_ was shown to be effective in reducing CCl_4_-induced liver fibrosis. In the LDH assay, rats administered *G. lucidum* (10, 30, and 100 mg/kg) showed better activity (Recknagel et al., 1974; Lin et al., 1995). The effect of *G. lucidum* extracts on GOT and serum lactate dehydrogenase showned that CCl_4_ combined with *G. lucidum* extracts significantly decreased liver damage in rats (Lin et al., 1995).

**TABLE 5:** Hepatoprotective effect of raw herbal extracts on CCl_4_-induced increase in GOT GPT levels.

Groups	Dose (mg/kg)	GOT	GPT	Protection (%)	LDH
Normal	—	120.83 ± 4.02	44.18 ± 2.45	—	—
CCl_4_	—	263.58 ± 8.11*	84.93 ± 2.29*	—	552.83 ± 57.58
GL	10	253.05 ± 16.26	77.07 ± 8.47	7.38	484.67 ± 70.13
	30	198.13 ± 19.32	64.37 ± 5.32	45.85	464.33 ± 22.40
	100	169.52 ± 14.82	52.68 ± 6.20	65.89	310.17 ± 100.41

Data source: (Lin et al., 1995) Significantly apart from the norm. *p < .001, Student’s t-test. Notably distinct from the CCl_4_-control group; % of protection: *p* = (C - 120.83) - (T - 120.83)/(C - 120.83); C, the GOT value of CCl_4_-controlled group; T, the GOT value of the drug-treated group.

GLE therapy significantly alleviated CCl_4_-induced living fibrosis, accompanied by increases in plasma transaminases, hepatic malondialdehyde and hydroxyproline (HP) levels, and decreases in plasma albumin A/G ratio and hepatoproteins (Lin and Lin, 2006). Additionally, GLE therapy lowered TGF-β1 expression and changed MAT1A and MAT2 expression. *G. lucidum* fermentation filtrate (FGL) was found to have the same pharmacological activity against CCl_4_-induced liver fibrosis (Kwon and Kim, 2011).

### 4.4 Protective effect of *G. lucidum* on non-alcoholic obesity-induced hepatic fibrosis

Non-alcoholic fatty liver disease (NAFLD) can result in severe fibrosis of the liver. Early detection and early treatment of NAFLD can significantly enhance therapy success rates (Zhou et al., 2021). Mice-fed high-fat food (HFD) showed signs of non-alcoholic steatosis, as evidenced by increased liver-to-body weight ratio, hepatic fat, and serum ALT levels. However, GL treatment was successful in ameliorating these abnormalities. An aqueous extract of GL effectively reduced obesity *via* modulation of the intestinal microbiota in rodents (Chang et al., 2015), as shown in Table 6. Other studies have reported that GL substrate extracts could effectively treat obesity by altering the expression of metabolic enzymes (Thyagarajan-Sahu et al., 2011).

**TABLE 6:** *G. lucidum* (GL) attenuates perirenal fat accumulation in the liver weight of mice fed a high-fat diet (HFD).

Groups	Liver weight (g)	Perirenal fat weight (g)
ND	1.25	0.62
ND + GL	1.05	0.59
HFD	1.92	1.66
HFD + GL	1.41	1.17

Data source: (Jung et al., 2018) Five times per week, GL (50 mg/kg) or a placebo was orally delivered to mice fed a normal diet (ND) or an HFD. Dietary consumption was assessed every 10 days for 16 weeks. After 16 weeks of GL, therapy, the mice were slaughtered and their tissues were weighed (*n* = 8–9 per group). The data is the mean.

In the liver, insulin resistance is associated with the amount of subcutaneous abdominal fat (Abate et al., 1995). Increased levels of cellular fatty acid derivatives stimulate stress kinases, resulting in the phosphorylation of insulin receptor substrate (IRS) proteins with serine (Capeau, 2008). A clinical investigation demonstrated that insulin resistance and hepatic steatosis are closely linked. Reduced glucose tolerance is indicative of insulin resistance and inappropriate glucose handling (Shulman et al., 1990). Glucose transporter protein 4 (GLUT4) plays a crucial part in glucose transport in muscle and adipose tissue (Zhao and Keating, 2007). Glucose translocation by GLUT4 is an insulin-dependent mechanism and a rate-limiting step in glucose consumption. According to the literature research in Table 7, after 6 weeks, GL therapy lowered fasting glucose levels and enhanced glucose and insulin sensitivity in HFD-fed rats. In addition, GL increased adipocyte GLUT4 protein levels. These findings imply that GL’s anti-adipogenic action may mitigate hyperglycemia (Jung et al., 2018).

**TABLE 7:** *G. lucidum* (GL) reduces fasting glucose levels, glucose tolerance, and insulin tolerance in HFD-fed mice.

Groups	Fasting blood glucose (mg/dl)	Glucose tolerance (mg/dL)	Insulin tolerance (mg/dL)
ND	90	122	166
ND + GL	98	116	169
HFD	168	218	247
HFD + GL	118	171	168

Data source: (Jung et al., 2018) In HFD-fed, animals, GL, decreased fasting blood glucose, glucose tolerance, and insulin tolerance. Oral administration of GL (50 mg/kg) or vehicle five times per week to mice fed a normal diet (ND) or a high-fat diet (HFD). Once every 2 weeks, the mice were fasted for 16 h to assess their blood glucose levels. At 14 weeks of GL, therapy, mice (*n* = 8–9) were fasted for 16 h to conduct the GTT, and ITT (*n* = 8–9 for each group). Statistics are the mean.

Cholesterol and triglycerides accumulate in liver cells, causing their deposition in the liver cells. They were generally considered the culprits of fatty liver (Zhai et al., 2008). Thus, in the context of steatosis, an excessive buildup of triglycerides (TG) inside the hepatocytes was released as very-low-density lipoprotein (VLDL), an essential precursor of LDL that possessed atherogenic features (Venkatesan et al., 1993; Jung et al., 2018). It was widely assumed that excessive liver production of VLDL contributed to numerous hyperlipidemic conditions in humans, such as familial combination hyperlipidemia and diabetes (Venkatesan et al., 1993). Total blood cholesterol (TC) and LDL levels were decreased in HFD-fed mice by GL. Consequently, the data revealed that GL may enhance the serum lipid profile and prevent the evolution of non-alcoholic steatosis. In conclusion, GL regulated energy metabolic processes and fat accumulation in the liver and adipocytes directly. It enhanced insulin sensitivity and metabolic problems in a diet-induced obese animal model. GL was a viable eligible for prevention or treatment metabolic disorders including NAFLD.

### 4.5 Protective effect of *G. lucidum* on formaldehyde-induced hepatic fibrosis

**FIGURE 5:**
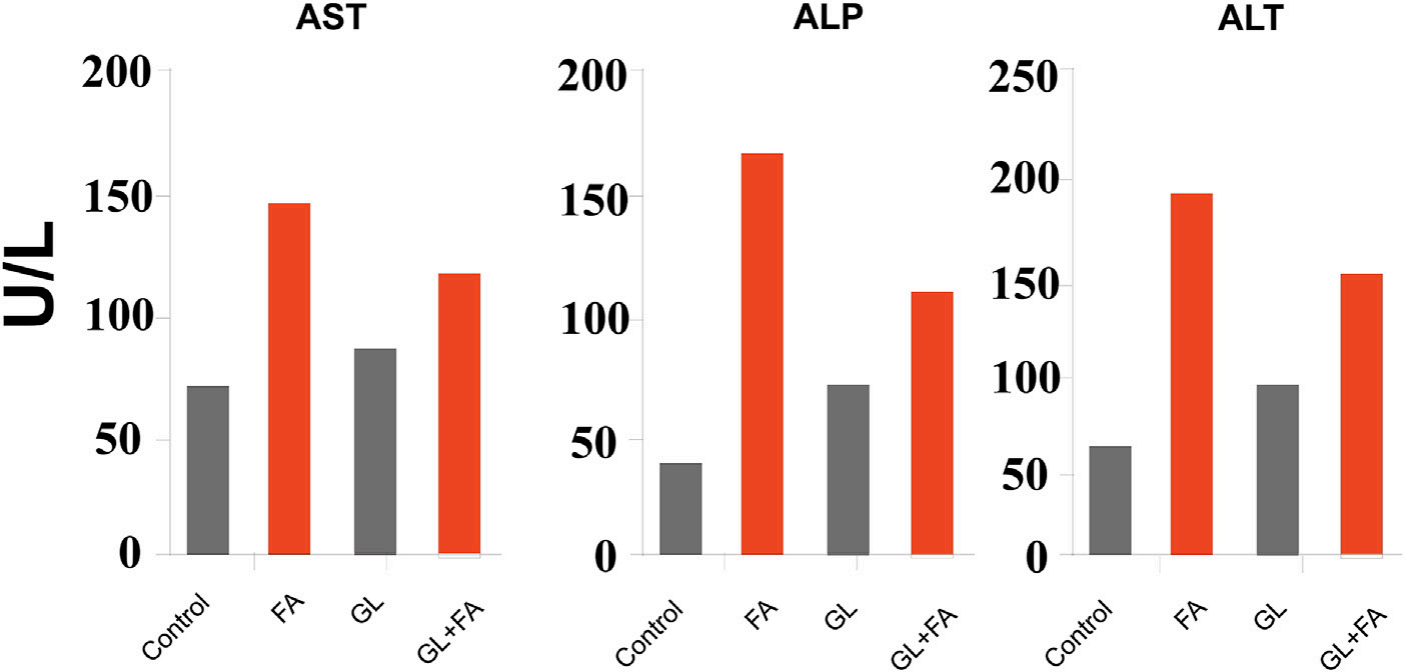
Data source: (Oluwafemi Adetuyi et al., 2020) formaldehyde and GL’s effect on hepatic enzyme markers. The data are displayed as the mean (*n* = 7). AST, aspartate aminotransferase; ALP, alkaline phosphatase; ALT, alanine aminotransferase.

*G. lucidum* extract exerts a preventive and therapeutic effect in experiments on liver fibrosis caused by formaldehyde (FA) exposure (Oluwafemi Adetuyi et al., 2020). By evaluating alanine aminotransferase (ALT), aspartate aminotransferase (AST), and alkaline phosphatase (ALP), the hepatoprotective efficacy of *G. lucidum* against FA-induced liver fibrosis was determined. ALT was an essential liver fibrosis enzyme responsible for catalyzing the transamination process. This increase in the number of enzymes will aggravate liver fibrosis (Kodavanti et al., 1989). AST and ALP were indicators of liver fibrosis; they were cytosolic and mitochondrial enzymes whose levels were frequently raised in the presence of persistent disease and necrosis resulting from lack of hepatocyte integrity. Such enzymes facilitated the exchange of α-amino acids from alanine and aspartate to the α-keto group of ketoglutarate, leading to the formation of pyruvate and oxaloacetate, respectively (Schiff et al., 2007). The FA group had considerably higher levels of these enzymes in contrast to the control group. Moreover, treatment with 100 mg/kg of *G. lucidum* considerably decreased the increased levels in Figure 5, demonstrating that *G. lucidum* protects rats from FA-induced liver fibrosis, as shown in Table 8. Ganoderma administration significantly reduced the elevated liver function enzymes (Lakshmi et al., 2006).

**TABLE 8:** Effects of *G. lucidum* and formaldehyde on the mean body weight and relative organ weight of rats.

Groups	Average body weight (g)	Relative organ weight (g)
Control	42.35 ± 4.52	7.32 ± 0.47
FA	18.75 ± 5.45	4.76 ± 0.78
GL	36.74 ± 6.32	8.76 ± 0.95
GL + FA	25.45 ± 6.32	6.75 ± 0.52

Data source: (Oluwafemi Adetuyi et al., 2020) Significantly different from control.

TNF, IL-1β, and IL-6 have a significant impact on the etiology of liver fibrosis. TNF is largely a set of pro-inflammatory cytokines recognized to play an essential part in inducing liver fibrosis, and there is evidence that oxidative stress and endotoxins may work together to promote TNF production (Feagins et al., 2008). Interleukins 1β and 6 are potential indicators of hepatotoxicity, either acute or chronic. The liver secreted pro-inflammatory cytokines TNF, IL-1β, and IL-6 into the bloodstream during hepatotoxic fibrosis. Consequently, biological therapies that block these cytokines demonstrated considerable therapeutic potential. When FA was delivered to rats, the levels of these cytokines were dramatically increased in the liver. A substantial decrease in cytokine levels was confirmed in the group administered 100 mg/kg of *G.lucidum*. These findings provide more evidence of the hepatoprotective action of *G. lucidum*. *G. lucidum* was able to counteract this impact, since the FA-treated rats exhibited extensive periportal cell infiltration and significant congestion (Batiha et al., 2020a; Oluwafemi Adetuyi et al., 2020).

This study showed that exposure to FA resulted in a significant decrease in anti-oxidant markers (Batiha et al., 2020b) and hepatic transaminases, triglycerides, and inflammatory markers increased. *G. lucidum* was ability to restore anti-oxidant, lipid, and anti-inflammatory status conferred a protective impact (El-Rahman et al., 2020).

## 5 Conclusion and perspective

*G. lucidum* has reached more than two thousand years of medicinal use in China and is also a traditional and valuable herb commonly used in our folklore, playing an important role in maintaining human health. The effective hepatoprotective activity of the natural active ingredients isolated from Ganoderma may represent an exciting advance in the search for effective hepatoprotective agents, particularly given the urgent need for the development of novel and innovative drugs as well as additional research, including clinical trials, to identify these natural compounds as good alternatives to conventional drugs. Therefore, most current studies on *G. lucidum* against liver fibrosis were conducted with the crude extract of *G. lucidum*. In the subsequent development, the components of *G. lucidum* can be purified and separated, and in the case of promising components, such as *G. lucidum* triterpenes can be finely separated. 1) The active components should be identified, and then performed cytotoxicity experiments. Subsequently, *in vitro* and *in vivo* experiments should be conducted to clarify the mechanism of action and the conformational relationships of its compounds. 2) We can optimize the scaffolds or moieties of natural drugs through synthetic reconstitution to stabilize or enhance their pharmacodynamic activities. 3) In the subsequent development of Ganoderma drugs, we can achieve the optimal therapeutic effect of the Chinese herbal formulas by using different ratios of ingredients based on the clear mechanism of the anti-liver fibrosis action of Ganoderma.

For example, the pharmacological effects of Ganoderma triterpenes are mainly focused on single components such as ganoderic acid A, ganoderic acid D, ganoderol F, or semi-purified components of the extract to analyze the pharmacological activities, followed by the ratio of various components in the triterpenes, i.e., the ratio of ganoderic acid to ganoderol, whether the two components have synergistic pharmacological effects, or the pharmacological effects of the ratio with traditional drug combinations are often neglected, i.e., at what ratio the anti-liver fibrosis activity is the strongest, which is not only in the development and utilization of single components of *G. lucidum*.

Therefore, these studies provide valuable insights and a certain working basis in the research of new drugs for *G. lucidum* against liver fibrosis. With the rising trend in the number of patients with liver fibrosis worldwide, there is a large market for effective drugs to treat liver disease. As a medicinal food source, *G. lucidum* is a drug with potential to be developed as an anti-liver fibrosis agent. The role of *G. lucidum* in maintaining liver function will be better applied and will receive more attention and application in the field of healthcare and pharmaceutical research, believing that it will make an important contribution to the human health industry.
